# Systematic Review of Pharmacological Properties of the Oligodendrocyte Lineage

**DOI:** 10.3389/fncel.2016.00027

**Published:** 2016-02-12

**Authors:** Carla Marinelli, Thomas Bertalot, Morena Zusso, Stephen D. Skaper, Pietro Giusti

**Affiliations:** Department of Pharmaceutical and Pharmacological Sciences, University of PaduaPadua, Italy

**Keywords:** oligodendrocyte, GABA, glutamatergic, cholinergic, muscarinergic, opioids, nuclear receptor, regeneration

## Abstract

Oligodendrogenesis and oligodendrocyte precursor maturation are essential processes during the course of central nervous system development, and lead to the myelination of axons. Cells of the oligodendrocyte lineage are generated in the germinal zone from migratory bipolar oligodendrocyte precursor cells (OPCs), and acquire cell surface markers as they mature and respond specifically to factors which regulate proliferation, migration, differentiation, and survival. Loss of myelin underlies a wide range of neurological disorders, some of an autoimmune nature—multiple sclerosis probably being the most prominent. Current therapies are based on the use of immunomodulatory agents which are likely to promote myelin repair (remyelination) indirectly by subverting the inflammatory response, aspects of which impair the differentiation of OPCs. Cells of the oligodendrocyte lineage express and are capable of responding to a diverse array of ligand-receptor pairs, including neurotransmitters and nuclear receptors such as γ-aminobutyric acid, glutamate, adenosine triphosphate, serotonin, acetylcholine, nitric oxide, opioids, prostaglandins, prolactin, and cannabinoids. The intent of this review is to provide the reader with a synopsis of our present state of knowledge concerning the pharmacological properties of the oligodendrocyte lineage, with particular attention to these receptor-ligand (i.e., neurotransmitters and nuclear receptor) interactions that can influence oligodendrocyte migration, proliferation, differentiation, and myelination, and an appraisal of their therapeutic potential. For example, many promising mediators work through Ca^2+^ signaling, and the balance between Ca^2+^ influx and efflux can determine the temporal and spatial properties of oligodendrocytes (OLs). Moreover, Ca^2+^ signaling in OPCs can influence not only differentiation and myelination, but also process extension and migration, as well as cell death in mature mouse OLs. There is also evidence that oligodendroglia exhibit Ca^2+^ transients in response to electrical activity of axons for activity-dependent myelination. Cholinergic antagonists, as well as endocannabinoid-related lipid-signaling molecules target OLs. An understanding of such pharmacological pathways may thus lay the foundation to allow its leverage for therapeutic benefit in diseases of demyelination.

## Introduction

**Figure 1:**
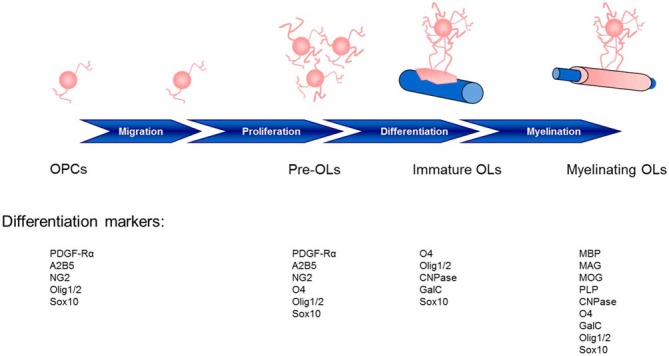
**Stages of oligodendrocyte maturation toward the oligodendroglial lineage: oligodendrocyte precursor cells (OPCs), preoligodendrocytes (pre-OLs), immature oligodendrocytes (OLs) and myelinating mature OLs.** These stages are identified by their increasingly complex morphology and expression pattern of well-defined markers. Two basic helix-loop-helix transcription factors, Olig1 and Olig2, play essential roles in determining the oligodendroglial lineage and in the generation and maturation of OLs (Zhou et al., 2000; Lu et al., 2001). The localization of Olig1/2 is observed in the nucleus of OPCs during development; however, although Olig2 remains in the nucleus of OPCs in the adult mouse (Arnett et al., 2004), Olig1 translocation into the cytosol highly correlates with the differentiation of OLs, the termination of the cell cycle and Olig1 phosphorylation (Niu et al., 2012). CNPase, 2′,3′-cyclic nucleotide 3′-phosphodiesterase; GalC, galactocerebroside C; MAG, myelin associated glycoprotein; MBP, myelin basic protein; MOG, myelin oligodendrocyte glycoprotein; PDGF-Rα, platelet-derived growth factor receptor; PLP, proteolipid protein.

The central nervous system (CNS) relies on a network of neuronal cells to transmit electrical impulses known as action potentials along the axon. A lipid-rich membrane, myelin (Virchow, 1854), insulates the axon and allows for rapid conduction of such impulses and proper delivery to the target cell (Hartline and Colman, 2007). Schwann cells supply myelin for the peripheral nervous system (PNS), whereas in the CNS, oligodendrocytes (OLs; Iglesias-Rozas and Garrosa, 2012) are responsible for myelin production, and are generated in the germinal zone from migratory bipolar oligodendrocyte precursor cells (OPCs; Grinspan, 2002; Brazel et al., 2003). Myelinating OLs not only provide trophic support for axons, but also release lactate through the monocarboxylate transporter 1 which is then utilized by axons for mitochondrial adenosine triphosphate (ATP) generation (Saab et al., 2013). The migration of OPCs is influenced by receptor-ligand adhesions with the extracellular matrix, such as integrins, and signaling molecules (Soliven, 2001) which may provide a critical link between neuronal cell activity and OPCs. Cells of the OL lineage acquire cell surface markers with maturation and respond specifically to factors which regulate proliferation, migration, differentiation, and survival (Figure 1).

**Figure 2:**
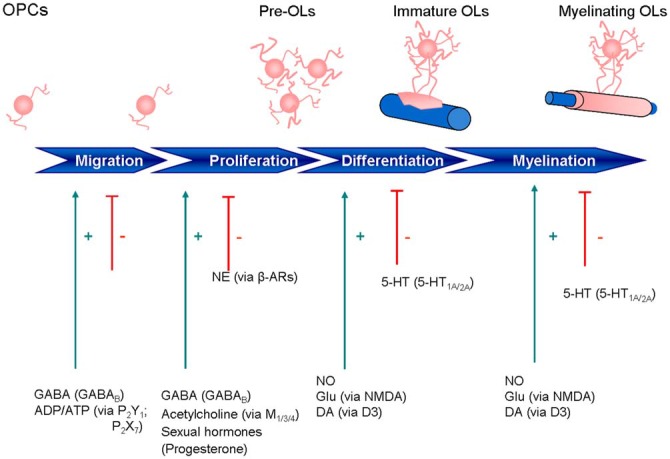
**Expression of neurotransmitter and other signaling receptors during oligodendrocyte maturation**. ADP, adenosine diphosphate; ATP, adenosine triphosphate; β-ARs, β-adrenoreceptors; DA, dopamine; GABA, γ-aminobutyric acid; GLU, glutamate; NE, norepinephrine; 5-HT, 5-hydroxytryptamine; NO, nitric oxide.

Impairment of one or more of these processes can lead to myelin degeneration, dysfunction or loss in nerve signal conduction and ultimately, nerve deterioration (Felts et al., 1997; Mensch et al., 2015). Loss of myelin results in a wide range of neurological disorders, including reduced motor function, impaired cognitive abilities, and vision problems. Demyelinating diseases of the CNS include multiple sclerosis (MS), acute disseminated encephalomyelitis, neuromyelitis optica, transverse myelitis, central pontine myelinolysis and leukodystrophy, while those of the PNS include chronic inflammatory demyelinating polyneuropathy, Guillain-Barré syndrome and Charcot-Marie-Tooth disease (http://www.neurodegenerationresearch.eu/about/what). Perinatal white matter (WM) injury, or periventricular leukomalacia, is the most common cause of brain injury in premature infants and is the leading cause of cerebral palsy. Late OPCs exhibit selective vulnerability in this last pathology (Back et al., 2007). Contrary to what was once believed, we now know that the mammalian CNS can undergo neurogenesis and gliogenesis, re-establishing axon-glial interactions needed for remyelination (Compston, 2002). Importantly, cells of the OL lineage express and respond to a broad range of receptor-ligand pairs, in particular neurotransmitters and nuclear receptors (NRs) like glutamate (Glu), γ-aminobutyric acid (GABA), ATP, serotonin, acetylcholine (ACh), nitric oxide (NO), opioids, prostaglandins, prolactin (PRL), cannabinoids and the superfamilty of NRs (steroid hormones, sex hormones, oxysterols, vitamin D3, thyroid hormone (TH), retinoic acid, fatty acid amides). Clearly, knowledge about the OL lineage has increased greatly over the past few years, with attention shifting towards manipulation of that lineage for therapeutic purposes. This review is intended to provide an overview of current knowledge on the pharmacological properties of the OL lineage, with particular attention to these receptor-ligand interactions and how they may influence OL migration, proliferation, differentiation, and myelination, together with an appraisal of their therapeutic potential. In this context, a number of mediators work through Ca^2+^ signaling, with the balance between Ca^2+^ influx and efflux determining the temporal and spatial properties of OLs. Moreover, Ca^2+^ signaling in OPCs can influence not only differentiation and myelination, but also process extension and migration, as well as cell death in mature mouse OLs. Research until now has identified cholinergic antagonists, as well as endocannabinoid-related lipid-signaling molecules with the ability to target OLs. Understanding the above-mentioned relationships may help to delineate additional new therapeutic avenues for remyelination/repair. The main systems examined are briefly summarized on Figure 2.

## Materials and Methods

**Figure 3:**
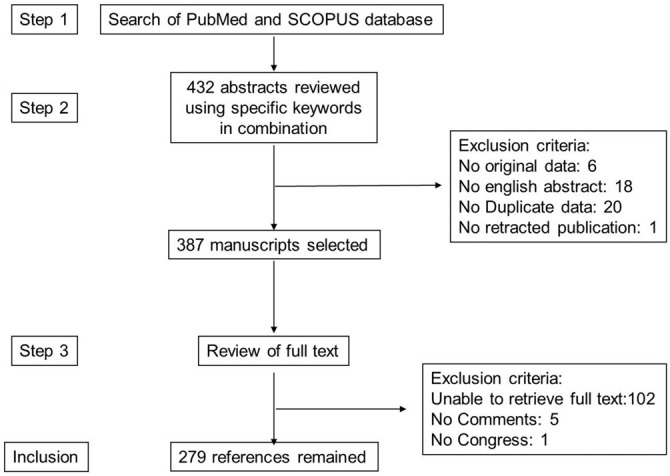
**Study inclusion flowchart**. This depicts the methodology for search and collection of relevant articles for this review, following PRISMA guidelines.

The literature search for this review was carried out according to the PRISMA (Preferred Reporting Items for Systematic Reviews and Meta-analyses) guidelines as they apply to systematic reviews (Moher et al., 2010). The search strategy is given after Conclusion and Perspectives. A total of 387 manuscripts were retrieved. Exclusion criteria are given in Figure 3. In all, 279 articles remained for this review.

### GABAergic Signals

GABA is the main inhibitory neurotransmitter in adult brain (Koós and Tepper, 1999; Markram et al., 2004; Takesian and Hensch, 2013), where it binds two main receptor types: fast hyperpolarizing ionotropic GABA_A_ receptors and metabotropic GABA_B_ receptors.

GABA participation in inhibitory neurotransmission is limited in adulthood, as in many brain regions during early development activation of GABA_A_ receptors excites neurons through activation of Na-K-Cl cotransporter1 (NKCC1) symporter which increases extracellular K^+^ and a depolarizing efflux of Cl^−^, in turn triggering Ca^2+^ influx (Dzhala et al., 2005). Similarly OPCs, but not OLs, express NKCC1. Muscimol, a GABA_A_ agonist, increases intracellular Ca^2+^ [Ca^2+^]_i_ protecting cultured OLs following withdrawal of culture medium supplements (N1, biotin, platelet-derived growth factor, PDGF; Wang et al., 2003). Using the GABA_A_ receptor antagonist pentylentetrazole, Mensch et al. (2015) showed a subtle increase in the number of OLs and a 40% increase in the number of myelin sheaths in the zebrafish ventral spinal cord. OLs may express also GABA_B_ receptors, whose activation increases proliferation and migration of OPCs via a negatively coupled adenlyl cyclase signaling pathway. The GABA_B_ receptor agonist baclofen significantly reduced cyclic adenosine monophosphate and adenlyl cyclase and led to an increase in OPC migration (Luyt et al., 2007).

### Glutamatergic Signals

Glu, the principal excitatory neurotransmitter, acts on postsynaptic ionotropic and G-protein-coupled metabotropic (mGluR) receptors. The former comprise the ligand-gated ion channel N-methyl-D-aspartate (NMDA), alpha-amino-3-hydroxy-5-methylisoxazole-4-propionic acid (AMPA) and kainate receptor subtypes (Dingledine et al., 1999; Swanson et al., 2005). These receptors are expressed by glial cells in both gray matter (Jabs et al., 2005; Matute, 2006; Bakiri et al., 2009) and WM (Matute, 2010). Virtually all neurons expressing ionotropic Glu receptors are sensitive to excitotoxic injury (Choi, 1988; Lipton and Rosenberg, 1994). Glu is toxic also for astrocytes (Haas and Erdö, 1991) and OLs (Matute et al., 1997; McDonald et al., 1998).

Cells of the OL lineage express functional AMPA and kainate-type Glu receptors throughout their development and across species, including man. NMDA receptors on both immature and mature OLs can be activated during injury (Káradóttir et al., 2005; Salter and Fern, 2005; Micu et al., 2006). OLs express as well all three groups of metabotropic Glu receptors in a developmentally-regulated fashion (Deng et al., 2004; Spampinato et al., 2015). In addition to astrocytes, OLs also express glutamate transporters, mainly the Glu aspartate transporter (GLAST; EAAT1 in the modern nomenclature). The neuronal transporter, termed excitatory amino acid carrier 1 (EAAC1; EAAT3 in the modern nomenclature), is present in a subpopulation of adult OPCs (Domercq et al., 1999).

Primary cells of the OL lineage are highly vulnerable to a form of excitotoxic injury mediated by a transporter-related mechanism involving inhibition of cystine uptake, glutathione depletion and oxidative stress (Oka et al., 1993). Alterations in Glu homeostasis in WM may thus determine injury to OLs and myelin. Prolonged activation of GluRs is toxic to primary OLs *in vitro* and *in vivo* (Matute et al., 1997; McDonald et al., 1998; Li and Stys, 2000).

Activation of AMPA and kainate receptors on microglia leads to the release of tumour necrosis factor-α (TNF-α), which can potentiate Glu neurotoxicity and kill OLs, destroy myelin and damage axons (Merrill and Benveniste, 1996). Inflammatory cytokines like TNF-α and interleukin-1β released by reactive microglia can impair Glu uptake and trigger excitotoxic OL death (Takahashi et al., 2003). Indeed, inhibiting the expression and function of Glu transporters in axonal tracts is sufficient to induce OL loss and demyelination (Domercq et al., 2005). AMPA receptors on OLs lack GluR2 subunits, suggesting a higher Ca^2+^ permeability than for these cells in gray matter (Matute, 2006).

Myelin regeneration can occur spontaneously, even in pathological conditions such as MS. Using an *in vivo* remyelination model, Gautier et al. (2015) demonstrated that demyelinated axons are electrically active and generate *de novo* synapses with recruited OPCs which, early after lesion induction, sense neuronal activity by expressing AMPA/kainate receptors. Furthermore, blocking neuronal activity, axonal vesicular release or AMPA receptors in demyelinated lesions results in reduced remyelination. In the absence of neuronal activity there is a ~6-fold increase in OPC number within the lesions and a reduced proportion of differentiated OLs. These findings reveal that neuronal activity and release of glutamate instruct OPCs to differentiate into new myelinating OLs that recover lost function (Gautier et al., 2015).

Another mechanism of Glu action on OPC differentiation involves activation of specific NMDA receptor subunits, as NMDAR1 and NMDAR2A protein levels increase during differentiation whereas NMDAR2B and NMDAR3 levels decrease (Sawada et al., 1996; Cavaliere et al., 2012). These authors showed that activation of NMDA receptors during OLs differentiation elevated cytosolic Ca^2+^ levels and promoted myelination when co-cultured with neurons. NMDA receptors on multipotent stem cells promote maturation of OLs and favor myelination through production of reactive oxygen species; levels of the latter correlate with degree of differentiation, an effect negatively modulated by the NADPH inhibitor apocynin (Cavaliere et al., 2012). Interestingly, NMDA receptors are expressed in clusters on OL processes, whereas AMPA and kainate receptors are diffusely located on the cell somata (Káradóttir et al., 2005; Salter and Fern, 2005; Micu et al., 2006).

Activation of mGlu4 on astrocytes appears to be involved in sparing OLs from excitotoxic challenge (Spampinato et al., 2015), hinting that they may be a novel target to protect from demyelination. Other pharmacological approaches, such as ionotropic Glu receptor antagonists, increase OL survival but have no effect on neuroinflammation (Pitt et al., 2000). A close interplay between astrocytes and OLs is supported by the observation that kainate-induced toxicity is attenuated by stimulation of mGlu4 receptors only in a mixed culture of OLs and astrocytes; the mGlu4 receptor agonist L-AP4 does not act directly on OLs. Activation of mGluRs, including mGlu4 on astrocytes, is reported to be neuroprotective (Yao et al., 2005; Corti et al., 2007).

Soluble factors released by astrocytes might mediate L-AP4-increased OL viability. Transforming growth factor beta 1, which increases upon L-AP4 treatment, protects OLs from kainate-induced toxicity (Spampinato et al., 2015), an effect attenuated by a neutralizing anti-transforming growth factor beta 1 antibody. Factors that contribute to Glu homeostasis include glutaminase, which is present in OLs and microglia (Domercq et al., 1999; Werner et al., 2001). Non-toxic concentrations of Glu acting at kainate receptors can induce OL death also by sensitizing these cells to complement attack (Alberdi et al., 2006), an action mediated by proteolysis of complement protein C5 to generate the potent chemoattractant C5a which interacts with its G-protein-coupled receptor C5aR (CD88; Ward and Newman, 1969) on OLs and other CNS cells (Nataf et al., 2001).

In conclusion, factors that evoke an increase in [Ca^2+^]_i_ can regulate cell proliferation, survival, growth and differentiation or induce a pathological condition, as a function of OL developmental stage.

### Purinergic Signals

ATP is a co-transmitter released with other “classical” transmitters and activates ionotropic (P2X) and metabotropic (P2Y) receptors (Ralevic and Burnstock, 1998; North, 2002). In the CNS, ATP can be released by neurons and glia via membrane transporters, vesicles, and ATP-permeable channels (Fields and Stevens, 2000; Ballerini et al., 2002; Coco et al., 2003). OPCs express several ionotropic (P2X_1,2,3,4,7_) and metabotropic (P2Y_1,2,4_) receptors, with P2X_7_ and P2Y_1_ receptors being the main ionotropic and metabotropic P2 receptors, respectively (Agresti et al., 2005). ATP and adenosine diphosphate (ADP) may regulate OPC functions through P2Y_1_ receptors. For example, ATP and ADP (the P2Y_1_-specific agonist ADPbS) induced OPC migration. Moreover, ATP and ADP inhibited OPC proliferation induced by PDGF in purified cell cultures and in cerebellar tissue slices (Agresti et al., 2005). These effects of ATP and ADP on cell migration and proliferation were being blocked by a P2Y_1_ antagonist (MRS2179). P2Y1 receptors localize to NG2-labeled OPCs in the developing rat brain (Agresti et al., 2005).

OPC migration involves the interactive effects of growth factors, chemokines, integrins, neurotransmitters, and extracellular matrix molecules (Decker et al., 2000; Tsai and Miller, 2002). PDGF and basic fibroblast growth factor induce OPC migration *in vitro*, acting mainly as chemotactic stimuli (Armstrong et al., 1990; Milner et al., 1997), while ATP enhances the migration of OPCs by stimulating predominantly random motility and to a lesser extent, chemotaxis (Agresti et al., 2005). These authors propose that ATP, acting mainly at P2Y_1_ receptors, may be important in stimulating OPC migration along a chemotactic gradient towards specific sites. However, under pathological conditions the P2X_7_ receptor is involved in OPC migration (Feng et al., 2015). High concentrations of ATP or the P2X_7_ receptor agonist BzATP increased the number of migrating OPCs *in vitro*, while pre-treatment with the P2X_7_ receptor antagonist oxidized ATP decreased the promotive effect. This effect appeared to depend on Fyn, a member of the Src family of kinases (Feng et al., 2015).

Differentiated OLs *in vitro* express functional P2X and P2Y receptors, which can act as mediators of axono-oligodendroglial communication during myelination (Alberdi et al., 2005). P2X_1_ and P2X_3_ receptors produce rapidly desensitizing currents whereas P2X_7_ receptors, as well as P2X_2_ and P2X_4_ receptors, may undergo a conformational change which results in formation of a large pore upon prolonged exposure to ATP (Khakh et al., 1999). ATP signaling can directly trigger OL excitotoxicity via activation of Ca^2+^-permeable P2X_7_ receptors, leading to WM lesions reminiscent of MS plaques (Walz et al., 1993; Butt, 2006).

### Serotoninergic Signals

Serotonin (5-hydroxytryptamine, 5-HT) plays critical roles in early neural development, being involved in neuronal cell differentiation and migration, axonal growth and pathfinding, dendritic arborization, synaptogenesis, circuit formation, and neuronal plasticity (Daubert and Condron, 2010). Mice with either 5-HT transporter (Esaki et al., 2005) or monoamine oxidase A gene deficiencies (Cases et al., 1996) show disrupted primary somatosensory cortical organization.

Depression is reported to be accompanied by myelin and OL abnormalities (Yamazaki et al., 2007), including a significant reduction in myelin basic protein (MBP) expression in multiple brain areas of post-mortem samples from depressed patients (Honer et al., 1999; Taylor et al., 2004; Regenold et al., 2007). Further, Aston et al. (2005) found in temporal cortex a down-regulation of both OL development-related and myelin formation-related genes.

Manipulation of 5-HT levels in postnatal day 6–8 rats with selective 5-HT reuptake inhibitors (SSRIs; Xu et al., 2004) or *p*-chlorophenylalanine-induced 5-HT depletion (Persico et al., 2000) leads to disorganized cortical barrel fields, abnormal social behavior, callosal myelin malformation, and OL pathology. 5-HT receptor subtypes 1A and 2A have been found in OL lineages and to co-localize with developmental lineage-selective markers (Fan et al., 2015). Exposure to 5-HT not only disturbed development of OLs with aberrant process outgrowth and reduced MBP and proteolipid protein (PLP) expression, but even caused a developmentally-dependent cell death at the highest doses via 5-HT_2A_. By means of a neuron-OL myelination co-culture model, Fan et al. (2015) demonstrated that 5-HT exposure alters the localization and regular space patterns of the paranodal contactin-associated protein (Caspr), but did not cause OL death or reduce OL density. These authors suggested that 5-HT exposure induces a diffuse and enlarged pattern affecting other axon-derived factors for myelination.

It is tempting to speculate that manipulation of 5-HT levels affects OL development and myelination, thereby contributing to the altered neural connectivity seen in SSRI-treated animals (Simpson et al., 2011). SSRIs (Loughhead et al., 2006) and serotonin/norepinephrine reuptake inhibitors (Wang et al., 2014), which are widely prescribed for major depression in pregnant women, are able to cross the placenta and reach fetal compartments. Animal data suggest that extracellular 5-HT levels in CNS could rise up to 20-fold upon SSRI exposure (Tao et al., 2000). Such high levels of extracellular 5-HT may adversely affect OL development and/or myelination, perhaps contributing to altered neural connectivity seen in depression and in autism spectrum disorders. Further, the late maturation of human myelin, especially in prefrontal cortex has potential implications in the treatment of humans with SSRIs in early adolescence or young adulthood.

Spinal cord injury (SCI) causes secondary damage involving OL cell death and axon demyelination. SSRIs like fluoxetine improve neurological outcome in animal models of SCI by inhibiting microglia activation which can cause the death of OLs. The underlying mechanism involves inhibition of p38 mitogen-activated protein kinase (p38-MAPK) and pro-nerve growth factor expression. In addition, fluoxetine can attenuate Ras homolog gene family member A activation, decrease the level of phosphorylated c-Jun and alleviate activation of caspase-3. Fluoxetine may thus be a novel therapeutic agent for acute SCI in humans (Lee et al., 2015).

### Catecholaminergic Signals

The brain’s principal catecholamine neurotransmitters include 3,4-dihydroxyphenethylamine (dopamine, DA) and 4,5-β-trihydroxyphenethylamine (norepinephrine, NE). DA controls locomotor activity, cognition, emotion, positive reinforcement, food intake, and endocrine regulation (Missale et al., 1998). Neuropsychiatric and motor function disorders like schizophrenia and Parkinson’s disease (PD) are often linked to abnormal DA signaling (Civelli et al., 1993). DA receptors can be divided into two main groups: D_1_-like (D_1_ and D_5_; Sunahara et al., 1991; Tiberi et al., 1991) and D_2_-like (D_2_, D_3_, D_4_; Sokoloff et al., 1990; Van Tol et al., 1991; Sawada et al., 1998).

D_2_ and D_3_ receptors are expressed by differentiated rat cortical OLs (Rosin et al., 2005), although little is known about their role(s) in OL biology. Cortical OLs *in vitro* are vulnerable to receptor-independent Glu-induced cell death (Oka et al., 1993; Rosin et al., 2004) which involves oxidative stress, inhibition of cystine uptake and depletion of intracellular stores of reduced glutathione (Murphy et al., 1990). Glu-induced death of cortical OLs was protected by co-treatment with either D_2_ or D_3_ agonists (Avenell et al., 1999) and appeared to involve quinpirole-sensitive activation of MAPK/extracellular signal-regulated kinase and oxidative stress (Rosin et al., 2005). The protective effect of quinpirole was fully attenuated by the D_2_ receptor antagonist L-741,626 (Avenell et al., 1998). Intriguingly, oxygen/glucose deprivation up-regulated expression of D_2_ and D_3_ receptors over a much shorter time scale (2 h) compared to the time needed to induce injury (18 h; Rosin et al., 2005). Cellular responsiveness to oxygen/glucose deprivation injury may depend on the state of OL maturation (Deng et al., 2003). As D_3_ receptors are expressed in differentiating OLs before terminal maturation, it is possible that DA plays a role in OL lineage differentiation and/or the formation of myelin by mature OLs (Bongarzone et al., 1998).

NE is a hormone and neurotransmitter that affects brain areas involved in vigilant concentration. NE underlies the fight-or-flight response, directly increasing heart rate, triggering the release of glucose from energy stores, and increasing blood flow to skeletal muscle. OPCs express functional α- and β-adrenoreceptors (ARs). Response to NE is enhanced following OPC maturation into differentiated OLs: α_1A_-ARs induce phosphoinositide hydrolysis without effecting OPC proliferation, while β-ARs increase intracellular cyclic AMP and inhibit their proliferation (Cohen and Almazan, 1993; Ghiani et al., 1999). The effects of NE can be mirrored by molecules like phorbol esters, which mimic the action of second messengers, leading to inhibition of terminal differentiation of OLs and block of myelin-specific protein expression (Baron et al., 1998). Protein kinase C activators work at a later stage of differentiation to facilitate myelination (Vartanian et al., 1986; Asotra and Macklin, 1993; Yong et al., 1994).

### Cholinergic Signals

ACh was the first identified neurotransmitter, and has important roles in neuron-glia signaling (Wessler et al., 2001). ACh can act on different tissues and cell types (Proskocil et al., 2004; Kawashima and Fuji, 2008) to modulate growth, survival, differentiation, and apoptosis (Eglen, 2005). While muscarinic cholinergic receptors can promote neuronal cell differentiation during neurogenesis (Lauder, 1993; Tata et al., 2003; Salani et al., 2009), nicotinic cholinergic receptors affect inflammation centrally by modulating microglial release of cytokines, and peripherally through immune system cells (Tracey, 2002; De Simone et al., 2005; Kawashima and Fuji, 2008). Several glial cell populations, including OLs express ACh receptors (Murphy et al., 1986; Van Der Zee et al., 1993; Rogers et al., 2001; Loreti et al., 2007).

Stimulation of muscarinic receptors on OLs (Larocca and Almazan, 1997; Molina-Holgado et al., 2003) activates MAPK and increases inositol trisphosphate levels and Ca^2+^ waves (Ritchie et al., 1987; Kastritsis and McCarthy, 1993; Cohen and Almazan, 1994; Larocca and Almazan, 1997). Rat OPCs express all muscarinic receptors with particular abundance of M_3_, while mature OLs have barely detectable levels of all subtypes except for M_2_, suggesting a dynamic regulation during OL maturation (De Angelis et al., 2011). Furthermore, specific physiological role(s) may be inferred from their diverse cellular distribution: M_1_ and M_3_ are localized in cell bodies and processes while M_4_ is found only in the cell body (De Angelis et al., 2011).

Muscarinic receptors can affect OPCs proliferation. For example, carbachol induces proliferation via both muscarinic and nicotinic cholinergic receptors (Peralta et al., 1987; Cohen et al., 1996). De Angelis et al. (2011) used selective muscarinic antagonists to discriminate receptor subtype involved in OPC proliferation. These authors showed that only M_1_, M_3_ and M_4_ antagonists were able to reduce proliferation. Indeed, gallamine did not affect proliferation via M_2_ antagonism. In contrast, M_2_ activation by arecaidine influenced OPC survival in a dose-dependent manner, showing toxic effects, indicating the involvement of M_2_ in OPC survival (De Angelis et al., 2011).

ACh receptors have been extensively studied in terms of expression levels of myelin proteins (i.e., MBP, PLP, and the myelin and lymphocyte protein). Muscarine treatment of cultured OPCs and mature OLs decreased expression of MBP (De Angelis et al., 2011), although the drug’s effect on expression of other myelin proteins was equivocal, suggesting a specific role of muscarinic activation in MBP production and terminal differentiation of OLs (De Angelis et al., 2011). However, it should be kept in mind that MBP is a marker for mature myelinating and non-myelinating OLs but not OPCs. Given their ability to control glial cell proliferation, M_1_, M_3_ and M_4_ muscarinic receptor antagonists (Felder et al., 2000; Wess, 2004; Langmead et al., 2008; McArthur et al., 2010) might be useful in the treatment of brain tumors such as gliomas and oligodendrogliomas (Guizzetti et al., 1996; Loreti et al., 2007; Tata and Calogero, 2010).

Muscarinic drugs could also find utility in stem cell therapy, based on their capability to regulate myelin repair in demyelinating diseases such as MS (Goldman et al., 2012). Abiraman et al. (2015), using OPCs isolated from human fetal forebrain showed that the potent, non-selective muscarinic receptor agonist oxotremorine-M reduces OL differentiation. In contrast, the selective M_3_ antagonist darifenacin (Moriya et al., 1999) failed to influence OL differentiation, suggesting that M_3_ receptors are not expressed by human OPCs and/or that darifenacin does not act as an inverse agonist. However, when human OPCs were cultured with fetal human neuronal re-aggregates, ACh release from ACh^+^ neurons delayed OPC differentiation in a darifenacin-sensitive manner, suggesting that M_3_ antagonism could promote human OL differentiation (Abiraman et al., 2015). Further, solifenacin, a blood-brain barrier-permeable analog of darifenacin, promoted differentiation and myelination in neonatal mice transplanted with human OPCs (Maruyama et al., 2008). Collectively, these findings encourage the view that such pharmacological treatments could one day serve to induce resident OPC differentiation and improve outcome in demyelinating diseases such as MS, where OL differentiation is impaired (Franklin and Kotter, 2008; Franklin and ffrench-Constant, 2008).

### Nitric Oxide

NO is a gaseous, cell-penetrant neurotransmitter. Under physiological conditions NO is present in the picomolar-nanomolar range in the CNS (Hall and Garthwaite, 2009), where it is generated by neuronal NO synthase (nNOS) in response to NMDA receptor-triggered rises in [Ca^2+^]_i_ (Hall and Garthwaite, 2009). NO stimulates specialized guanylyl cyclase-coupled receptors to generate cyclic GMP (Garthwaite, 2008; Hardingham et al., 2013). Neurons and astrocytes are the principal targets of NO, although neuronally-derived NO may also act on the microvasculature to regulate blood flow (Yang et al., 2003). NO, superoxide, and hydrogen peroxide regulate MBP phosphorylation which contributes to neuron–oligodendrocyte signaling by promoting stability of the myelin sheath (Atkins and Sweatt, 1999).

The cerebellum is an important area for motor control and one in which NO-cyclic GMP signaling contributes to synaptic plasticity and other phenomena (Garthwaite, 2008; Contestabile, 2012). Myelination in the rat cerebellum begins in the central WM at around postnatal day 6 (P6) and proceeds along the axial core of the lobules to appear in the internal granule cell layer 4–6 days later (Gianola et al., 2003). The development of OL NO-reactivity between P3 and P8 would be consistent with its role in this process. In cortical cell cultures, NO and cyclic GMP enhance arborization of OLs. Compounds that increase NO activity promote OL growth and maturation (Garthwaite et al., 2015). Moreover, NO can coordinate axon myelination with neuronal activity during development and contribute to adaptive changes in adult myelination (Garthwaite et al., 2015). However, in OLs high NO concentrations can inhibit mitochondrial respiration and contribute to oxidative/nitrosative stress through peroxynitrite formation that induces uncontrolled MBP phosphorylation (Atkins and Sweatt, 1999), which may render the myelin sheet less stable. Therefore, anti-oxidant and mitochondria protective therapeutic strategies may be beneficial in MS, in particular in early stages of the disease (Lassmann, 2011).

### Opioids

Neural stem cells (NSCs) and cells at different stages along the oligodendrolial lineage express opioid receptors, whose modulation can induce both mitogenesis and differentiation (Knapp et al., 1998; Persson et al., 2006; Eschenroeder et al., 2012). These cells are also capable of synthesizing opioids in a developmentally regulated manner (Knapp et al., 2001). Cultured bipolar progenitors produce dynorphin, whose expression is lost upon differentiation into mature OLs. In contrast, proenkephalin-derived peptides are found in OPCs and differentiated OLs (Knapp et al., 2001). Activation of mu opioid receptor (MOR) in cultured OPC stimulates DNA synthesis, whereas kappa opioid receptor inhibition increases membrane extensions (Knapp et al., 1998). Although these observations suggest that interference with the endogenous opioid system affects OL maturation and myelination in the developing CNS, further studies will be needed to fully resolve this question.

The growing use/abuse of opioids among younger persons (Compton and Volkow, 2006; Calcaterra et al., 2013) poses an especially high risk to pregnant addicts (in whom drug effects could target early myelination in the fetus and newborn) and adolescents and young adults (in whom late myelination of cortical regions takes place). Prolonged administration of morphine to neonatal rats is associated with increased neuronal cell apoptosis in selective CNS areas (Bajic et al., 2013) and leads to increased Toll-like receptor-4 and microglial activation in adolescent rats (Schwarz and Bilbo, 2013). Heroin and morphine abusers can suffer from leukoencephalopathy and myelin loss (Chen et al., 2000; Nanan et al., 2000). Perinatal exposure to therapeutic doses of methadone and buprenorphine (used to treat opioid addiction) altered early myelination in the developing rat brain (Eschenroeder et al., 2012; Vestal-Laborde et al., 2014), with elevated brain levels of MBP, PLP, and myelin-oligodendrocyte glycoprotein. Not surprisingly, these animals showed an increased number of axons with a highly compacted myelin membrane. There was no parallel reduction in the number of axons with a still uncompacted myelin membrane, pointing to the existence of complex drug effects. Methadone exerts direct effects at specific stages of the OL lineage *in vitro*, stimulating the proliferation of OPCs while accelerating maturation of more differentiated but still immature preOLs (Vestal-Laborde et al., 2014). While the long-term effects of these observations remain unknown, accelerated or increased OL maturation and myelination could disrupt the complex sequence of synchronized events leading to normal connectivity in the developing brain. These findings further point to a crucial function of the endogenous opioid system in controlling OL development and the timing of myelination, as well as the potential for disrupting brain maturation at critical stages of myelin formation.

The partial MOR agonist and kappa opioid receptor antagonist buprenorphine merits special comment. While treatment of mothers with therapeutic doses of methadone results in accelerated and increased brain expression of mature markers of OLs in pups, supra-therapeutic doses delayed MBP expression and decreased the number of myelinated axons (Eschenroeder et al., 2012). Buprenorphine anti-nociceptive action also showed a biphasic bell-shaped curve, with low doses being analgesic and higher doses loosing efficacy (Dum and Herz, 1981; Lizasoain et al., 1991). Buprenorphine anti-nociceptive effects are mediated by MOR and counteracted by nociceptin (NOP) receptors (Lutfy et al., 2003). In the latter study J-113397, a specific NOP receptor inhibitor, not only enhanced the anti-nociceptive effect of buprenorphine but also eliminated its bell-shaped response. This biphasic response was not seen in NOP receptor knockout mice (Lutfy et al., 2003). In OL cultures J-113397 abolished the biphasic dose-dependent effect of buprenorphine on MBP expression (Eschenroeder et al., 2012). The latter findings point to a crucial role for interactive MOR and NOP receptor-mediated signaling in the timing of OL maturation. Perinatal exposure to buprenorphine increases the caliber of myelinated axons and with disproportionally thinner myelin sheaths, possibly indicative of alterations in axon-oligodendroglia/myelin interaction (Sanchez et al., 2008). Exposure of rat pups to the MOR agonist methadone did not cause these effects (Vestal-Laborde et al., 2014).

### Prostaglandins

Cyclooxygenase (COX) catalyzes the rate-limiting step in the synthesis of prostanoids from arachidonic acid (Smith et al., 1996), and occurs as both constitutive (COX-1) and inducible (COX-2) forms (Hewett et al., 2000). COX-2 expression increases in CNS neurons in response to GluR activation (Nogawa et al., 1997; Carlson, 2003), and its inhibition by non-steroidal anti-inflammatory drugs and non-selective COX-1/COX-2 inhibitors protects neurons from GluR-mediated excitotoxic death *in vitro* (Hewett et al., 2000; Carlson, 2003) and *in vivo* (Nogawa et al., 1997). Transgenic mice over-expressing neuronal COX-2 are more susceptible to excitotoxicity (Kelley et al., 1999) and age-associated neuronal cell loss (Andreasson et al., 2001). In contrast, COX-2-deficient mice are more resistant to neuronal cell death caused by ischemia or NMDA receptor activation (Iadecola et al., 2001).

OLs and OPCs are more susceptible to excitotoxic death following GluR-dependent COX-2 induction (Carlson et al., 2010). COX-2 is expressed in dying OLs at the onset of demyelination in Theiler’s murine encephalomyelitis virus model of MS (Carlson et al., 2006) and in dying OLs in MS lesions (Carlson et al., 2010). In the former case COX-2 inhibitors reduced demyelination (Carlson et al., 2010). COX-2 contributes to OL vulnerability also in the cuprizone model of demyelination (Palumbo et al., 2012). COX-2 may thus have an important role in demyelinating diseases like MS.

Prostanoids can contribute to excitotoxic death in OPCs either via direct production by OPCs or activation of prostanoid receptors on OPCs. Prostaglandin E_2_ (PGE_2_) synthesized by OPCs in a GluR-dependent fashion engages EP3 subtype receptors and contributes to excitotoxic death of OPCs (Carlson, 2003). In contrast, the EP2 receptor is protective (Carlson, 2003). Although EP1 receptor activation does not affect an OPC excitotoxic challenge (Carlson et al., 2015), PGI_2_ can promote OPC migration and remyelination in damaged areas (Takahashi et al., 2013). During myelination in developing rat brain, EP3 and thromboxane A2 receptor are localized to the plasma membrane and perinuclear membrane of OPCs, and their expression levels—as well as thromboxane A2 synthesis—increase during OLs maturation in concert with enhanced MBP expression (Carlson et al., 2010).

Given the role of EP3 receptors in OPC excitotoxic death, this receptor may be a potential therapeutic target for promoting remyelination in demyelinating diseases such as MS.

### Prolactin

PRL, originally described in the anterior pituitary gland plays a role not only in reproduction, but also controls a variety of behaviors and may participate in cell homeostasis, as well (Kelly et al., 1991; Freeman et al., 2000). Initially described in the brain in hypothalamic axon terminals (Fuxe et al., 1977), PRL immunoreactivity was later detected in telencephalon, cerebral cortex, hippocampus, amygdala, septum (Devito, 1988), caudate putamen (Harlan et al., 1989), brain stem (Devito, 1988; Harlan et al., 1989), cerebellum (Seroogy et al., 1988), spinal cord (Harlan et al., 1989; Siaud et al., 1989), choroid plexi, and circumventricular organs (Thompson, 1982). The PRL receptor belongs to class 1 of the cytokine receptor superfamily (Bazan, 1990a,b) whose gene shows widespread CNS expression.

PRL increases neurogenesis in the maternal mouse forebrain during the first week of pregnancy, along with an increase in OPC proliferation, OL generation, MBP expression and ultimately, increased numbers of myelinated axons (Gregg et al., 2007). PRL infused subcutaneously can mimic these effects (Gregg et al., 2007). These observations suggest a novel form of WM plasticity in the adult CNS and propose that PRL may have therapeutic potential in treating WM damage. At the same time, several reports suggest that PRL can exert also an inflammatory role. In an animal model of MS, the D2 agonists bromocriptine and dihydroergocryptine reduced the PRL level and disease severity (Riskind et al., 1991; Canonico et al., 1993; Zhornitsky et al., 2015). Although PRL or PRL receptor knockout mice develop a delayed onset experimental autoimmune encephalomyelitis but with full clinical severity (Costanza et al., 2013) a small clinical trial failed to demonstrate benefit of bromocriptine administration in MS patients (Bissay et al., 1994). Thus, the therapeutic utility of PRL in MS remains an open question; it would be interesting to perform a trial combining PRL with an immunomodulator (i.e., interferon-β commonly used in clinical practice; Zhornitsky et al., 2015).

### Cannabinoids

Cannabidiol (CBD), the most abundant cannabinoid in *Cannabis sativa*, is devoid of psychoactive properties and exerts anti-inflammatory, antioxidant and neuroprotective effects (Izzo et al., 2009). CBD is used for the treatment of inflammation, pain and spasticity associated with MS (Sastre-Garriga et al., 2011). Its biological effects are mediated through cannabinoid receptors CB_1_ and CB_2_ (Howlett et al., 2002). The former is widely expressed in CNS neurons, where it modulates neurotransmitter release and synaptic plasticity regulated by Ca^2+^ and K^+^ channels (Chevaleyre et al., 2006; Basavarajappa, 2007). CB_2_ receptor expression is mainly, but not exclusively limited to immune cells (Walter and Stella, 2004). OPCs and mature OLs both expess CB_1_and CB_2_ receptors (Molina-Holgado et al., 2003; Benito et al., 2007).

In rat optic nerve OLs, the CB_1_ receptor agonist arachidonoyl-chloro-ethanolamide inhibited depolarization-induced Ca^2+^ influx (Mato et al., 2009). Similar effects were obtained with the endocannabinoids anandamide and 2-arachidonoylglycerol and the CB_1_ receptor agonist CP 55,940, but not the CB_2_ receptor agonist JWH133. In line with these findings CBD, which acts also as a low-affinity CB_1_/CB_2_ receptor antagonist raised [Ca^2+^]_i_ concentration in OL primary cultures (Mato et al., 2009, 2010) and was cytotoxic (Mato et al., 2010). However, these [Ca^2+^]_i_ changes appeared to be CB receptor-independent (Mato et al., 2010). Given its lipophilic properties, it is possible that CBD can directly affect [Ca^2+^]_i_ stores. Not only are mitochondria the main source of Ca^2+^ released from CBD-stimulated OLs (Mato et al., 2010), but CB_1_ receptors have been identified on brain mitochondrial membranes (Hebert-Chatelain et al., 2014). In contrast, in neurons CBD reduced [Ca^2+^]_i_ influx and prevented apoptosis (Ryan et al., 2009). The patho-physiological consequences of CBD action may thus depend on the cellular context in which it is expressed.

### Nuclear Receptors

NRs are members of a large superfamily of evolutionarily related transcription factors that regulate a broad spectrum of physiological phenomena (Gronemeyer et al., 2004; Chambon, 2005; Evans, 2005). Each of the 48 NRs identified to date has crucial and non-redundant roles, notably in biologically important processes like growth, development, and homeostasis. NRs modulate transcription through mechanisms which include both activation and repression (Germain et al., 2006b). The NR superfamily includes receptors for hydrophobic molecules such as steroid hormones (e.g., estrogens, glucocorticoids (GCs), progesterone, mineralocorticoids, androgens, vitamin D3, ecdysone, oxysterols and bile acids), retinoic acids, TH, fatty acids, leukotrienes and prostaglandins (Laudet and Gronemeyer, 2002).

#### Steroid Hormone Receptors

GCs are steroid hormones with recognized anti-inflammatory properties. In the brain, two types of high-affinity receptors bind GCs: the type I mineralocorticoid receptor (MR) and the type II glucocorticoid receptor (GR; Vielkind et al., 1990). Each receptor dimerizes with a GC-responsive element leading to suppression of pro-inflammatory gene expression via block of nuclear factor-kB or activator protein-1 (Trousson et al., 2009). Both receptor types are widely expressed across neuronal cell populations and glia, although the latter finding awaits confirmation. Purified OLs and astrocytes from rat cerebrum and cerebellum were shown to express differential levels of GR by immunocytochemistry (Vielkind et al., 1990). At least *in vitro*, all classes of glial cells express GRs that can translocate to the nucleus in the presence of the cognate hormone ligand (Vielkind et al., 1990).

Dexamethasone (DEX) alters OLs differentiation and myelination in a developmental stage-specific manner. In rat forebrain cell cultures DEX stimulated the early stages of myelination but caused a markedly inhibition at later stages (Almazan et al., 1986). When given systemically to 3-day-old rats for 7 consecutive days DEX significantly decreased the relative abundance of MBP and PLP mRNAs at P20 and P30 in cerebrum, although in cerebellum MBP, PLP and glial fibrillary acidic protein mRNAs decreased at P10 (Tsuneishi et al., 1991). DEX thus appears to suppress expression of genes related to glial functions, especially myelination, when administered in the early postnatal period (Tsuneishi et al., 1991), notwithstanding differences in susceptibility across brain areas.

#### Sex Hormones

Sexual dimorphism in the brain vocal control area of the songbird was first demonstrated four decades ago (Nottebohm and Arnold, 1976). Magnetic resonance imaging shows morphological differences in human cortical gray matter volume (Gur et al., 1999) with females having larger volumes in areas associated with language functions (Schlaepfer et al., 1995; Harasty et al., 1997). In terms of WM differences between sexes, men have larger overall WM relative to cerebral size (Goldstein et al., 2001), while women appear to have larger WM volumes involved in interhemisphere connectivity (Nopoulos et al., 2000). Sexual dimorphism in rats was interpreted as a consequence of the action of different sex hormones on brain development (Cerghet et al., 2009).

We know relatively little concerning sexual dimorphism of OLs and central myelin. WM volume (Gur et al., 1999), especially in the corpus callosum (Fitch et al., 1990) is increased in males, with females having more unmyelinated fibers. Yang et al. (2008) later confirmed that WM volume, myelinated fiber volume and volume of myelin sheaths in young rats are significantly larger in males, although this ratio was reversed in middle-aged animals. Differences in steroid hormone levels in developing animals could account for sex differences in myelination of axons through hormone action on myelin protein expression (Jung-Testas and Baulieu, 1998; Nuñez et al., 2000). It is also possible that sex steroids could exert a protective effect on myelin in developing, as well as injured WM (De Nicola et al., 2006; Gerstner et al., 2007; Garay et al., 2008).

Sexual differences in rodents are also manifested in terms of OL myelin protein expression (Cerghet et al., 2009). Female rodent OLs showed increased proliferation and death rate, suggesting that their OLs have a shorter lifespan and more rapid turnover. In male mice, castration increased glial cell proliferation and decreased OL density (Cerghet et al., 2009). Sex hormones, in particular testosterone, influence proliferation and are potent inducers of remyelination (Hussain et al., 2013). In enriched mouse OLs primary cultures, progesterone (P2), estrogen (E2) and dihydrotestosterone (DHT) influenced total cell number, proliferation and cell death independent of sex. In particular, P2 significantly increased OL numbers (more so in cultures from female mice), while E2 had a minor effect and DHT reduced OL numbers in both sexes (Cerghet et al., 2009).

Protein kinase B (Akt), MAPK and mammalian target of rapamycin (mTOR) pathways are highly expressed in OLs (Flores et al., 2000; Horiuchi et al., 2006) and represent, in relation to cell survival/proliferation, possible biochemical targets for these hormones (Hay, 2005; Chong et al., 2007). P2 caused a more robust Akt up-regulation in OLs cultured from female mice in comparison to male-derived cells, in line with effects of P2 on OL numbers. Akt influences cell survival and proliferation through downstream mTOR activation via transcriptional regulation (Hay, 2005; Chong et al., 2007). P2 markedly increased mTOR activity in an Akt-dependent manner, indicating a role for this pathway in OL survival. As expected from its effects on OL numbers, DHT reduced Akt activity in both sexes (Cerghet et al., 2009). Interestingly, MAPK p42/p44 activation is involved in excitotoxic cell death, mediated by Glu and pro-inflammatory processes and leading to neurodegenerative diseases (Rosin et al., 2004; Nikodemova et al., 2006). It is tempting to speculate that by influencing proliferation and death of Ols, sex hormones may contribute to differences in disease phenotype between male and female MS patients.

#### Oxysterols and Liver X Receptors

The liver X receptor (LXR) is a member of the NR family of transcription factors (Janowski et al., 1996) and is closely related to NRs such as the peroxisome proliferator-activated receptor (PPAR), farnesoid X receptor and retinoid X receptor (RXR). Both LXR isoforms (α and β) are activated by oxysterols originating from cholesterol oxidation (Freemantle et al., 2013). OLs express LXR α and β which are crucial for cholesterol homeostasis, a major lipid constituent of myelin sheaths. LXRs are involved in myelination and remyelination processes. LXR_α/β_ double knockout mice exhibit altered motor coordination and spatial learning, thinner myelin sheaths, and reduced myelin gene expression in cerebellum. Conversely, activation of LXRs by either 25-hydroxycholesterol or the agonist TO901317 stimulates myelin gene expression at the promoter, mRNA, and protein (PLP and MBP) levels. LXR activation also promotes OLs cell maturation and remyelination after lysolecithin-induced demyelination in organotypic cerebellar slice cultures. These data strongly support a new role for LXRs as positive modulators in central (re)myelination processes (Meffre et al., 2015).

#### 1,25-Dihydroxyvitamin D3

1,25-Dihydroxyvitamin D3 (1,25(OH)2D3) was initially described to suppress experimental autoimmune encephalomyelitis (Lemire and Archer, 1991; Spach et al., 2006) through an anti-inflammatory mechanism. New data show that 1,25(OH)2D3 can directly promote neural stem cell (NSC) proliferation, survival and differentiation along the neuron and OL, but not astrocyte, lineages. NSCs constitutively express vitamin D receptor, which can be up-regulated by 1,25(OH)2D3. NSCs treated with 1,25(OH)2D3 showed increased expression of neurotrophic factors subserving neuronal cell survival and differentiation (Shirazi et al., 2015).

#### Retinoic Acid and Retinoid X Receptor

The RXR (Prineas and Connell, 1979) is activated by 9-cis retinoic acid (Patani et al., 2007), as well as 9-cis-13,14-dihydro-retinoic acid (Raine and Wu, 1993) and comprises three isoforms: RXR-α, -β and -γ. RXRs work through heterodimeric association with other NRs, such as retinoic acid receptors, TH receptors, vitamin D receptors, PPARs and LXRs to regulate cell proliferation, differentiation and apoptosis (Germain et al., 2006a). RXR-γ is generally expressed at low levels in all glial cell populations (Moreno et al., 2004) but is up-regulated in activated microglia and macrophages, reactive astrocytes and OLs after CNS injury (Schrage et al., 2006). RXR signaling is associated with the CNS regenerative response and is involved in the remyelination transcriptome (Huang et al., 2011). The remyelination environment is composed of demyelinated axons, activated adult OPCs, regenerated OLs, microglia and/or macrophages and reactive astrocytes (Franklin and ffrench-Constant, 2008). Functional analyses in cultured OPCs using RNA interference to knock down RXR-γ gene expression, RXR-specific antagonists, as well as RXR-γ null mice showed inefficient OL differentiation, indicating that RXR-γ is an important regulator of remyelination (Huang et al., 2011). The ability of RXR to form permissive or non-permissive heterodimers with other NRs suggests that RXR can modulate the expression of different genes, in a receptor- and context-specific fashion (Altucci et al., 2007). Identification of which nuclear receptor(s) heterodimerize with RXR-γ in OL lineage cells after demyelination, and what genes are transcribed in response to RXR-γ activation to promote the differentiation of OPCs have remained open questions until now. However, a new study by de la Fuente et al. (2015) provides data indicating that RXR-γ binds to several NRs, including the vitamin D receptor, in OPCs and mature OLs. These authors showed that inhibition of vitamin D receptor signaling impaired OPC differentiation and reduced the cells’ ability to remyelinate axons *ex vivo*. Vitamin D, in contrast, boosted OPC differentiation (de la Fuente et al., 2015).

#### Thyroid Hormone

TH actions are mediated mainly by T3 (Yen, 2001) which derives by conversion of T4 to T3 via deiodinases. THs activate two nuclear hormone receptor genes. The THR-α gene encodes two isoforms (α1 and α2) while four additional isoforms are encoded by the β gene (β1–4; Williams, 2000; Yen, 2001; Tagami et al., 2010). THs play a critical role in oligodendrogenesis and myelination. Acting through their cognate receptors, THs promote expression of OL-specific genes including MBP, myelin-associated glycoprotein (MAG), PLP, and 2′,3′-cyclic nucleotide 3′-phosphodiesterase (CNPase) (Farsetti et al., 1991; Tosic et al., 1992; Rodriguez-Pena et al., 1993). Studies using hypo- and hyperthyroid animals as well as genetically modified rodents clearly show that THs regulate OL differentiation and maturation (Bernal, 2002; O’Shea and Williams, 2002; Zoeller and Rovet, 2004; Dugas et al., 2012). *In vitro*, OPCs express both THR-α and THR-β1; expression of the latter increases with OL maturation (Barres et al., 1994; Gao et al., 1998). GC-1, a thyromimetic with selective THR-β action (Chiellini et al., 1998), promotes differentiation of both rodent and human OPCs *in vitro* (Trost et al., 2000; Manzano et al., 2003). Furthermore, GC-1 induces OL maturation during development with increased production of MBP, CNPase and MAG (Baxi et al., 2014).

OL differentiation can follow several distinct pathways, including mitogen withdrawal and exposure to T3 (Barres et al., 1993; Dugas et al., 2010), whereas OPC proliferation is induced by PDGF (Barres et al., 1992). When exposed to T3 and PDGF, OPCs continue to proliferate, differentiating only after multiple rounds of cell division (Temple and Raff, 1986; Barres et al., 1994; Durand and Raff, 2000). Like T3, GC-1 does not significantly affect PDGF-induced OPC proliferation. Inappropriate OL differentiation could thus deplete OPC numbers and hinder myelination (Baxi et al., 2014).

#### PPAR-γ and 15-deoxy 12,14 Prostaglandin J2

The three known PPARs (PPAR-α, PPAR-β/δ and PPAR-γ) belong to the superfamily of NRs. While ubiquitously expressed, they also exhibit specific patterns of distribution. PPAR-γ agonists exert anti-inflammatory, insulin-sensitizing and neuroprotective effects, as well as promote the protection and differentiation of OLs (Bernardo et al., 2009). In addition, PPAR-γ agonists increase OL mitochondrial respiratory chain activity and the cell’s ability to respond to environmental signals with oscillatory Ca^2+^ waves. Both OL maturation and oscillatory Ca^2+^ waves are prevented by the mitochondrial inhibitor rotenone and restored by PPAR-γ agonists, suggesting that PPAR-γ promotes myelination through mechanisms involving mitochondria (Bernardo et al., 2009, 2013). Calcium signaling plays an important role in OPC differentiation and myelination (Soliven, 2001), process extension and migration (Simpson and Armstrong, 1999; Yoo et al., 1999), and in retraction of membrane sheets and cell death in mature mouse OLs (Benjamins and Nedelkoska, 1996).

## Conclusion and Perspectives

The main functions of OLs in CNS are to provide support and insulation to axons of some vertebrates, forming functional units that allow the propagation of electrical signals. On the other hand, neuronal activity regulates myelinating cell development through signals that activate Ca^2+^-dependent pathways (Butt, 2006). As discussed in this review, activation is evoked by a wide range of membrane/intracellular receptors or ion channels (for the latter, see Cheli et al., 2015) that can induce an influx or release from intracellular stores, increasing [Ca^2+^]_i_. To restore homeostasis, [Ca^2+^]_i_ can be sequestered into endoplasmic reticulum or extruded (Butt, 2006). The balance between Ca^2+^ influx and efflux can determine the temporal and special properties of OLs. Moreover, Ca^2+^ signaling in OPCs can influence not only differentiation and myelination (Soliven, 2001), but also process extension and migration (Simpson and Armstrong, 1999; Yoo et al., 1999), and cell death in mature mouse OLs (Benjamins and Nedelkoska, 1996). There is also evidence that oligodendroglia exhibit Ca^2+^ transients in response to electrical activity of axons for activity-dependent myelination (Wake et al., 2015).

Failure of myelin development and oligodendrocyte loss results in serious human disorders, including MS. Understanding how different neurotransmitters and hormones can regulate [Ca^2+^]_i_, driving important effects on different stages of OPCs development and specific molecular events could provide the framework to facilitate the development of effective new approaches in the therapeutic repair of myelin disorders that complement established immunosuppressive approaches. In this regard the landscape is beginning to show the fruits of these investigations (Mullard, 2014; Ransohoff et al., 2015). For example, several U.S. Food and Drug Administration-approved drugs have shown promise in this direction, although most are anticholinergics. Whether or not the other possible pharmacological pathways are ineffective or have not been tried in not known. The antidyskinetic drug benztropine, a selective M1 muscarinic acetylcholine receptor antagonist, induced MBP expression in primary rat optic-nerve-derived OPCs and decreased clinical severity in an experimental autoimmune encephalomyelitis model of relapsing-remitting MS when administered alone or in combination with the approved immunosuppressive MS drugs FTY720 or interferon-β (Deshmukh et al., 2013). However, potential dose-dependent side-effects are associated with benztropine treatment in man (Modell et al., 1989). Systemic treatment with the selective M3 antagonist solifenacin increased oligodendrocyte differentiation of transplanted human OPCs in hypomyelinated shiverer/rag2 brain and enhanced functional repair (Abiraman et al., 2015). Donepezil, an acetylcholinesterase inhibitor developed for the treatment of Alzheimer’s disease, can stimulate oligodendrocyte differentiation and maturation of NSC-derived OPCs (Imamura et al., 2015). Importantly, neural precursor cells have recently been demonstrated to make a major contribution to remyelination in the cuprizone model of CNS demyelination (Xing et al., 2014). Although some of the reviewed pathways are involved in apoptosis (e.g., opioids, acetylcholine), the PPAR-α agonist palmitoylethanolamide (a fatty acid amide signaling molecule structurally related to the endocannabinoid anandamide), presented as a co-ultramicronized composite with the flavonoid luteolin is also reported to promote OPC maturation (Barbierato et al., 2015). Neither palmitoylethanolamide (Skaper et al., 2014) not luteolin (Theoharides et al., 2012), at pharmacologically relevant doses, has been reported to show adverse effects in man. While we have focused on MS as an emblematic pathological condition that could eventually benefit from OL-based repair therapies for myelin loss, preventive treatment of premature infants with drugs that accelerate the proliferation and/or maturation of OPCs could reveal itself as the key to prevent an important cause of mental retardation. No doubt the initial results discussed in this review will encourage further progress in the years ahead.

## Search Strategy

A search for original articles focusing on OLs and neurotransmitters and signaling was performed in PubMed and SCOPUS. The search terms used were “oligodendrocyte”, “myelin”, “myelination”, “oligodendrocyte precursor”, “nuclear receptor”, “opioid”, “glutamate”, “GABA”, “serotonin”, “cholinergic”, “muscarinic”, “cannabinoid”, “demyelination”, “autoimmune”, alone and in combination (other terms?). All articles identified were English language (with the exception of Virchow, R. 1854, originally written in German), full-text articles. We also searched the reference lists of identified articles for additional relevant articles.

## Author Contributions

PG and CM conceived the study, and PG, CM, TB, MZ and SDS participated in drafting the manuscript. All authors critically revised and approved the final manuscript.

## Conflict of Interest Statement

The authors declare that the research was conducted in the absence of any commercial or financial relationships that could be construed as a potential conflict of interest.
